# The RNA Replication Site of Tula Orthohantavirus Resides within a Remodelled Golgi Network

**DOI:** 10.3390/cells9071569

**Published:** 2020-06-27

**Authors:** Katherine A. Davies, Benjamin Chadwick, Roger Hewson, Juan Fontana, Jamel Mankouri, John N. Barr

**Affiliations:** 1 School of Molecular and Cellular Biology, University of Leeds, Leeds LS2 9JT, UK; katherine.davies@phe.gov.uk (K.A.D.); bmbmch@leeds.ac.uk (B.C.); J.Fontana@leeds.ac.uk (J.F.); j.mankouri@leeds.ac.uk (J.M.); 2 National Infection Service, Public Health England, Porton Down, Salisbury SP4 0JG, UK; Roger.Hewson@phe.gov.uk

**Keywords:** hantavirus, Tula virus, replication, factory, RNA synthesis, Golgi, stress granules

## Abstract

The family *Hantaviridae* within the *Bunyavirales* order comprises tri-segmented negative sense RNA viruses, many of which are rodent-borne emerging pathogens associated with fatal human disease. In contrast, hantavirus infection of corresponding rodent hosts results in inapparent or latent infections, which can be recapitulated in cultured cells that become persistently infected. In this study, we used Tula virus (TULV) to investigate the location of hantavirus replication during early, peak and persistent phases of infection, over a 30-day time course. Using immunofluorescent (IF) microscopy, we showed that the TULV nucleocapsid protein (NP) is distributed within both punctate and filamentous structures, with the latter increasing in size as the infection progresses. Transmission electron microscopy of TULV-infected cell sections revealed these filamentous structures comprised aligned clusters of filament bundles. The filamentous NP-associated structures increasingly co-localized with the Golgi and with the stress granule marker TIA-1 over the infection time course, suggesting a redistribution of these cellular organelles. The analysis of the intracellular distribution of TULV RNAs using fluorescent in-situ hybridization revealed that both genomic and mRNAs co-localized with Golgi-associated filamentous compartments that were positive for TIA. These results show that TULV induces a dramatic reorganization of the intracellular environment, including the establishment of TULV RNA synthesis factories in re-modelled Golgi compartments.

## 1. Introduction

The genus *Orthohantavirus*, within the *Hantaviridae* family of the *Bunyavirales* order, comprises over 36 species of enveloped segmented negative stranded RNA viruses [1] that are found throughout the globe, with many capable of causing devastating human disease [2]. Orthohantaviruses segregate into either New World (NW) and Old World (OW) clades based on their country of isolation, with viruses of the OW clade being widespread throughout Eurasia, whereas NW viruses are found within the Americas. Orthohantaviruses are mostly associated with a specific rodent host, in which they cause persistent infections [3]. In contrast, many OW and NW orthohantaviruses are associated with pathologically distinct disease outcomes in humans. Typically, viruses of the OW clade, such as the Hantaan virus (HTNV) and the Seoul virus (SEOV), are associated with a haemorrhagic fever with renal syndrome (HFRS), whereas NW viruses including Andes virus (ANDV) and Sin Nombre virus (SNV) are the causative agents of hantavirus cardiopulmonary syndrome (HCPS). Both syndromes are characterised by extensive vascular leakage, with human mortality rates ranging from 0.1–10% for HFRS to around 40% for HCPS [4]. In rodent hosts, few cytopathic effects are apparent and the animals develop asymptomatic or subclinical persistent infections [5]. This outcome is mirrored in cell culture systems that become persistently infected [6], reflecting the ability of hantaviruses to evade pathogen surveillance and innate immune defences.

The orthohantavirus genome comprises three virion-associated negative sense RNA segments (vRNAs) named small (S), medium (M) and large (L) based on their relative sizes [7]. The S segment encodes the nucleocapsid protein (NP), which coats the vRNAs forming helical ribonucleoprotein (RNP) complexes that act as templates for RNA synthesis. The M segment encodes a glycoprotein precursor (GPC) that is processed by cellular proteases into Gn and Gc proteins that form envelope spikes with primary roles in virus attachment and entry. The L segment encodes the large (L) protein that is the catalytic component of the RNA-dependent RNA polymerase (RdRp), responsible for two distinct RNA synthesis activities; mRNA transcription to yield a single 5′ capped mRNA from each vRNA template, and RNA replication to amplify the three vRNA segments via complementary (cRNA) intermediates. Hantavirus transcription involves the acquisition of short 5′ capped oligoribonucleotides from host cell mRNAs by an RdRp-associated endonuclease, which are then used by the RdRp to prime mRNA synthesis using a ‘prime-and-realign’ mechanism [8]. The source of these primers for hantaviruses is thought to be processing bodies (PBs), that are non-enveloped cytoplasmic RNP granules where non-sense mRNAs are stored awaiting degradation, and with which the SNV NP has been shown to closely associate [9]. NP is thought to bind the mRNA cap and protect it from degradation by the PB resident de-capping enzymes including DCP1a and DCP2, host factors that are required for other members of the order *Bunyavirales* [10].

The replication of hantaviruses, as with all bunyaviruses, is thought to occur within cytoplasmic viral factories [11]. Specifically, the replication factory of the prototypical bunyavirus, Bunyamwera virus (BUNV) has been visualized and described in detail. In mammalian cells, BUNV establishes large virus factories around the Golgi stack, recruiting organelles, cytoskeletal proteins and other cellular factors that together form elongated membranous structures known as viral tubes [12]. These tubes are lined with multiple membrane-bound polymerase molecules that are the sites of viral RNA synthesis, subsequently leading to the generation of RNPs for virion assembly. RNPs are thought to travel to adjacent assembly sites in the Golgi with the aid of actomyosin motors [12], where they interact with the viral glycoproteins and bud into the Golgi lumen. Virions subsequently mature as they traffic within Golgi-derived vesicles to the plasma membrane for release. BUNV replication and assembly in insect cells, in which the virus establishes a persistent infection, similarly occurs in the Golgi [13]. However, it was found that in mosquito cells, viruses do not accumulate intracellularly and exit the cell immediately after assembly, with NP and L proteins accumulated in non-membrane-bound cytoplasmic aggregates [13].

In contrast to BUNV, the precise intracellular site of hantavirus replication has not been defined. As NP is the major protein component of RNPs, its location is expected to coincide with sites of RNA synthesis and previous work has shown that NP from the NW hantaviruses, ANDV and Black Creek Canal virus (BCCV), accumulates within membranous structures surrounding the Golgi [14,15]. Consistent with this, NP from the OW hantavirus Tula virus (TULV) localizes in perinuclear regions [16] and HTNV NP resides within both perinuclear regions and the endoplasmic reticulum (ER)–Golgi intermediate compartment [17,18]. Further clues as to the location of sites of hantaviral RNA synthesis were revealed by the finding that the GFP-tagged L protein of TULV co-localized with the Golgi [19] and that a recombinant L protein from ANDV accumulated in perinuclear regions, co-localizing with both its cognate NP and also PB marker DCP1a [20]. Overall, with some notable NW hantavirus exceptions [21,22], hantavirus assembly is thought to occur by budding into the Golgi [7], suggesting the above description of BUNV replication and assembly sites may also broadly apply to those of hantaviruses.

In this study, we used confocal immunofluorescence microscopy alongside fluorescent single molecule in-situ hybridization (FISH) and electron microscopy (EM) to identify and characterise the cellular sites where NP as well as S segment RNAs were localised throughout an extended infection time course of the model OW hantavirus, TULV. We revealed that at peak and persistent stages of infection, NP is present as large filamentous and tubular structures, co-localizing with TIA-1, a marker for stress granules (SGs), and the Golgi, and that the FISH analysis strongly suggested that these TIA-1-containing and Golgi-derived structures are the sites of TULV RNA synthesis.

## 2. Methods

### 2.1. Virus and Cells

Tula virus (TULV) strain Moravia/5302v/95 was obtained from The National Collection of Pathogenic Viruses, PHE, UK. Stocks were shown to be mycoplasma free using MycoAlert (Lonza group AG, Basel, Switzerland). Vero E6 cells are a clone of Vero 76 cells isolated from *Cercopithecus aethiops* (African green monkey) kidney cells, and were obtained from the European Collection of Authenticated Cell Cultures (ECACC 85020206). These cells were selected as previous findings show that TULV-infection allows the establishment of a persistently infected state [23]. Virus titres were calculated by indirect staining using the anti-NP antibody in conjunction with an Alexa Fluor 488 secondary antibody (Thermo Fisher Scientific, Waltham, MA USA) as previously described [23]. 

### 2.2. Antibody Production and Validation

Antisera specific for TULV NP were generated in house as previously described [23], using bacterially expressed SEOV NP core domain as an antigen, purified as previously described, and used to immunize a sheep (AltaBiosciences, Redditch, UK). Antibodies were validated by Western blot analysis. 

### 2.3. TULV Infections of Vero E6 Cells

Briefly, Vero E6 cells were plated out at 1 × 10^6^ cells per 25 cm^2^ flask or at 1 × 10^5^ cells/well of a 12-well plate onto glass coverslips, sterilised by submerging into 100% ethanol and washing with sterile PBS. Cells were maintained in a humidified incubator at 37 °C with 5% CO_2_. TULV infections were carried out at a multiplicity of infection (MOI) of 0.1–0.5 in serum-free DMEM. Virus was adsorbed for 90 min before the addition of DMEM supplemented with 2% foetal bovine serum, 100 U/mL penicillin and 100 µg/mL streptomycin. For long-term infections, TULV infections were carried out in 25 cm^2^ flask and seeded onto coverslips 24 h prior to fixation. Fixation was carried out using 4% paraformaldehyde at 36 h post infection, 7 days post infection and 30 days post infection to represent early, peak and persistent infections.

### 2.4. Indirect Immunofluorescence

Mock-infected (media alone) or TULV-infected monolayers on coverslips were permeabilized in ice-cold methanol before washing in 1X PBS and blocked in 5% BSA in 1×PBS for 30 min. Primary staining was carried out using the following antibodies and reagents: in house anti-TULV NP [23], Golgi (Santa Cruz Biotechnology Inc, Dallas, TX AE6, USA), ER (Thermo Fisher Scientific Concanavalin A), β-actin (New England Biolabs (NEB), Ipswich, MA, USA D6A8), β-tubulin (NEB 9F3), vimentin (NEB D21H3), Rabs 5, 7 and 11 (NEB 2143, D95F2, D4F5), LAMP1 (NEB D2D11), clathrin heavy chain (NEB 4796), TIA-1 (Santa Cruz G-3) and DCP1a (Santa Cruz 56-Y). Incubations were carried out for 2 h at room temperature, followed by thorough washing in 1X PBS. Secondary antibody staining was carried out using donkey anti-goat Alexa Fluor 488 (Thermo Fisher Scientific A-11055), donkey anti-rabbit Alexa Fluor 555 (Thermo Fisher Scientific A-31572), donkey anti-mouse Alexa Fluor 555 (Thermo Fisher Scientific A-31570), donkey anti-rabbit Alexa Fluor 647 (Thermo Fisher Scientific A-31573), donkey anti-mouse Alexa Fluor 647 (Thermo Fisher Scientific A-31571) at a 1:500 dilution for 1 h. Nuclear staining was carried out by incubating with 300 nM DAPI for 3–5 min at room temperature. Cells to be visualised by confocal microscopy were mounted onto slides using Vectashield (Vector laboratories, Burlingame, CA, USA) hard set anti-fade mounting media and cured for 15 min before storing at 4 °C overnight. Cells to be further analysed by RNA-FISH were fixed in 4% formaldehyde for 20 min and stored in 1×PBS supplemented with 0.1 % diethylprocarbonate (DEPC).

### 2.5. TEM Preparation of TULV-Infected Vero E6 Cells

Vero E6 cells were fixed by adding 5% glutaraldehyde (*v/v* in 0.1 M phosphate buffer (PB) Agar Scientific, Stansted, UK) to an equal amount of cell media in the tissue culture flask, resulting in a final glutaraldehyde concentration of 2.5% (*v/v*). Cells were left for 2.5 h to fix at room temperature. After fixation, the cells were removed from the tissue culture flask by scraping and washed in 0.1 M PB for 30 min, twice. The cells were then post-fixed in 0.1 M PB with 1% osmium tetroxide (*w/v*) (Agar Scientific) for 1 h followed by four washes in 0.1 M PB. Subsequently, the post-fixed cells were dehydrated using an ascending ethanol series (40%, 60%, 80% and 2×100%) for 20 min, and then twice in propylene oxide (Agar Scientific) for 20 min. Dehydrated cells were incubated with a 1:1 mixture of propylene oxide and araldite epoxy resin (araldite CY212, dodecenyl succinic anhydride, and tri-dimethylaminomethyl phenol (Agar Scientific)) overnight, after which the mixture was replaced by pure araldite and incubated for 8 h. To ensure adequate embedding, new resin was added four times, after which the cells were polymerized at 60 °C for 24 to 48 h. 

Embedded cells were then sectioned in the ultra-thin range (80 to 100 nm) using a EM UC7 Ultramicrotome (Leica, Wetzlar, Germany). Ultra-thin sections were then placed upon 200 mesh carbon-coated copper-formvar grids and stained with lead citrate and uranyl acetate (UA). Sectioned cells were stained in 8% saturated (*w/v*) UA (Agar Scientific) for 1 h. UA-stained grids were then washed in dH_2_O for 5 min (×5). Afterwards, the sections were stained with lead citrate (Agar Scientific) for 10 min and washed in 0.02 M NaOH for 1 min (×3), and in dH_2_O for 1 min (×5). Sections were then imaged at 120 kV using a FEI Tecnai G2-Spirit electron microscope (Thermo Fisher Scientific) with a Gatan US4000/SP 4k × 4k CCD camera (Gatan, Pleasanton, CA, USA).

### 2.6. Fluorescent In-Situ Hybridisation (FISH) Probes

Fluorescent in-situ hybridisation DNA probes were designed against the sense and anti-sense TULV S segment ORF sequence using the Stellaris Probe Designer version 4.2 (LGC Biosearch Technologies, Novato, CA, USA). The Stellaris Probe Designer provided 48 oligonucleotide probe sequences that were 20 nucleotides in length with a minimum spacing of 2 nucleotides. Target TULV S segment ORF sequence was obtained from sequencing viral RNA isolated from TULV-infected cell culture medium (Genewiz Inc, South Plainfield, NJ, USA). Probes were assessed for cross-hybridization using NCBI Nucleotide Blast against *C. aethiops* (taxid:9534) mRNAs and against the TULV M and L segment sense and anti-sense sequences (strain: Tula/Moravia/5302v/95). Any probes with more than 15 consecutive complimentary nucleotides were discarded. TULV S segment sense and anti-sense probes were labelled with Quasar670, and all probe sequences are shown Table S1. Strand sets were kept separate throughout this study.

### 2.7. Fluorescent In-Situ Hybridisation (FISH)

Probe hybridisation was carried out according to manufacturers’ instructions. Briefly, each set of strand-specific FISH probes were reconstituted to 12.5 mM in RNase free 1× TE buffer (10 mM Tris-HCl, 1 mM EDTA pH 8.0 (G Biosciences) diluted in UltraPure DNase/RNase-Free Distilled water (Thermo Fisher Scientific) and stored separately at −20 °C. For each coverslip, 1 µL probe stock was used per 100 µL hybridisation buffer (Stellaris RNA FISH hybridisation buffer diluted with deionised formamide (Severn Biotech Ltd, Kidderminster, UK) in a 9:1 ratio). 

Cells were incubated in Stellaris RNA FISH wash buffer A diluted with deionized formamide and UltraPure DNase/RNase-Free Distilled water in a 2:7:1 ratio for 5 min at room temperature. Additionally, 100 µL hybridisation buffer with probes was dispensed into a droplet onto parafilm in a humidified chamber. Coverslips were incubated cell-side down onto probe mix for 4 h at 37 °C, protected from light. Cells were incubated in 1 mL Stellaris RNA FISH wash buffer A for 30 min at 37 °C, protected from light and then incubated in 1 mL Stellaris RNA FISH wash buffer A with 300 nM DAPI for 5 min at room temperature. The buffer was removed and the cells were incubated in 1 mL Stellaris RNA FISH wash buffer B for 5 min before mounting onto glass microscope slides using Vectashield hard-set antifade mounting media (Vector laboratories). Slides were cured for 45 min at room temperature, protected from light, and imaged within 24 h.

### 2.8. Quantitative RT-PCR

TULV RNA was isolated from infected cells or supernatant using the QIAamp viral RNA mini kit (Qiagen, Hilden, Germany), and the quantitative reverse transcriptase real-time PCR was carried out using the 1-step RT-qPCR kit (Promega, Madison, WI, USA), both as previously described [23]. For each qRT-PCR experiment, in vitro transcribed TULV S segment RNAs were diluted to generate 10^8^, 10^6^, 10^4^, 10^2^ genome copies/5 μL and used to build a standard curve from which the sample relative genome copies per mL could be calculated, as previously described [23].

### 2.9. Inhibition of Intermediate Filaments

Vero E6 were infected with TULV at a MOI of 0.5 in a 6-well plate. Infected cells were harvested for immunofluorescence and q-RT PCR analysis at 30 days post infection. Prior to harvesting, the cells were treated with the following cytoskeletal inhibitors; 17 μM nocodazole (Sigma-Aldrich, St. Louis, MO, USA) for 60 min and 400 nM okadaic acid (OA) (Sigma-Aldrich) for 30, 60 or 90 min. Infected Vero E6 cells treated with OA were also recovered by removing the inhibitor and incubating overnight with fresh media before harvesting. Mock treatments were carried out using an equal volume of solvent (dimethyl sulfoxide (DMSO)/ethanol). 

### 2.10. Laser Scanning Confocal Microscopy

Laser scanning confocal imaging was carried out using the Zeiss LSM700 confocal microscope using the 40×/1.3 Oil DIC Plan Apochromat, M27 objective lens with the diode 405 nm, 488 nm, 55 nm and 639 nm lasers or the Zeiss LSM880 + Airyscan upright confocal microscope using the Plan-Apochromat 40×/1.4 Oil DIC objective lens with the diode 405 nm, argon 488 nm, DPSS 561 nm and HeNe 633 nm lasers. Image analysis was carried out in Fiji Is Just ImageJ (FIJI).

## 3. Results

### 3.1. TULV NP Forms Both Punctate and Tubular Structures during Infection

**Figure 1:**
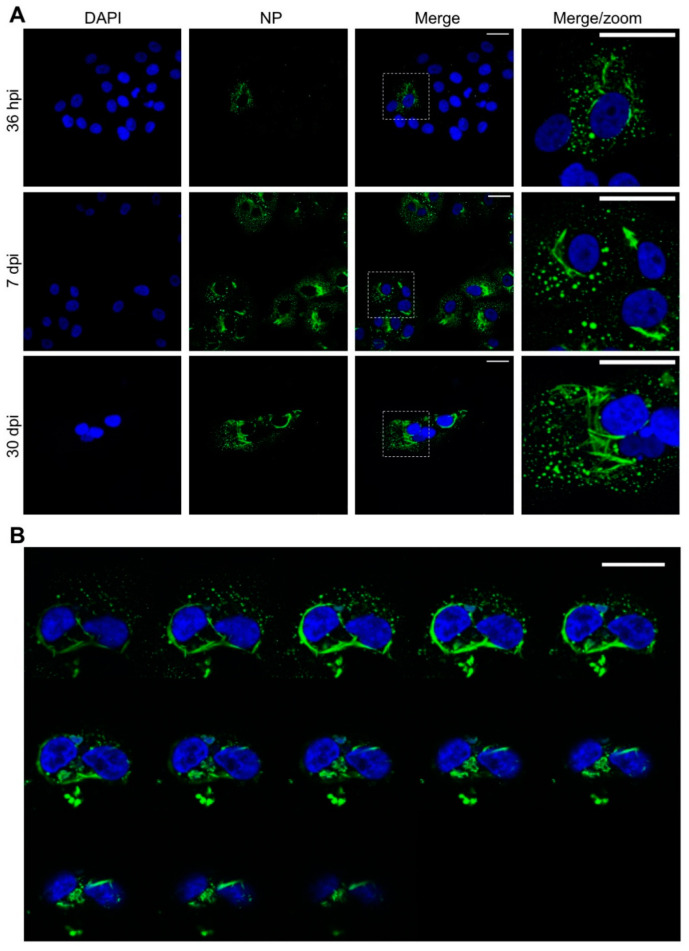
Confocal images showing the characteristic morphology of Tula virus (TULV) nucleocapsid protein (NP) -stained structures at early, peak and persistent stages of TULV infection in Vero E6 cells. (**A**) Vero E6 cells were infected with TULV at a multiplicity of infection of 0.1, and the images were taken at 36 h post infection (hpi), 7 days post infection (dpi) and 30 dpi using 40× magnification. Nuclei were stained with DAPI (blue) and TULV NP, detected using NP antisera (green), revealing the characteristic NP punctate and filamentous/tubular structures in the perinuclear region. (**B**) Successive Z sections of TULV-infected cells at 30 dpi show NP filamentous/tubular structures extending around the nucleus. The scale bars represent 30 μm.

We first analysed the intracellular distribution of TULV NP during an extended time course of up to 30 days, with time points of 36 h post infection (hpi), 7 days post infection (dpi) and 30 dpi chosen to represent the early, peak and persistent stages of TULV replication, respectively, as previously reported [23]. At the 36 hpi time point, NP mostly localised within discrete perinuclear puncta, with a minor component forming larger filamentous and tube-like structures (Figure 1A, top row). At this early time point, NP compartments had an average area of 0.54 µm^2^ (14 cells and 1248 compartments analysed) with the largest structure within a single focal plane having an area of 11.8 µm^2^. After 7 dpi, the NP compartments had an average area of 0.96 µm^2^ (31 cells and 2959 compartments analysed) with the largest structure having an area of 154 µm^2^ (Figure 1A, middle row). At 30 dpi, when fewer cells were present within the culture, but when surviving cells were still producing infectious TULV [23], most of the NP was present as tubes/filaments, reaching a maximum area of 513 µm^2^ (15 cells and 8615 compartments analysed), with only low levels of punctate NP visible (Figure 1A, bottom row). Across the time course, the length of the filamentous/tubular structures increased, and their diameters remained relatively constant (Figure 1A).

To reveal further information of the three-dimensional morphology of the large NP-stained structures, successive Z axis sections of persistently infected cells (0.25 µm per stack) were reconstructed to a generate a three-dimensional image. These revealed that the long tubular and filamentous structures were connected and appeared to wrap around the nucleus (Figure 1B and Movie S1).

### 3.2. Imaging of TULV-Induced Filamentous Cytoplasmic Structures Using Transmission Electron Microscopy

**Figure 2:**
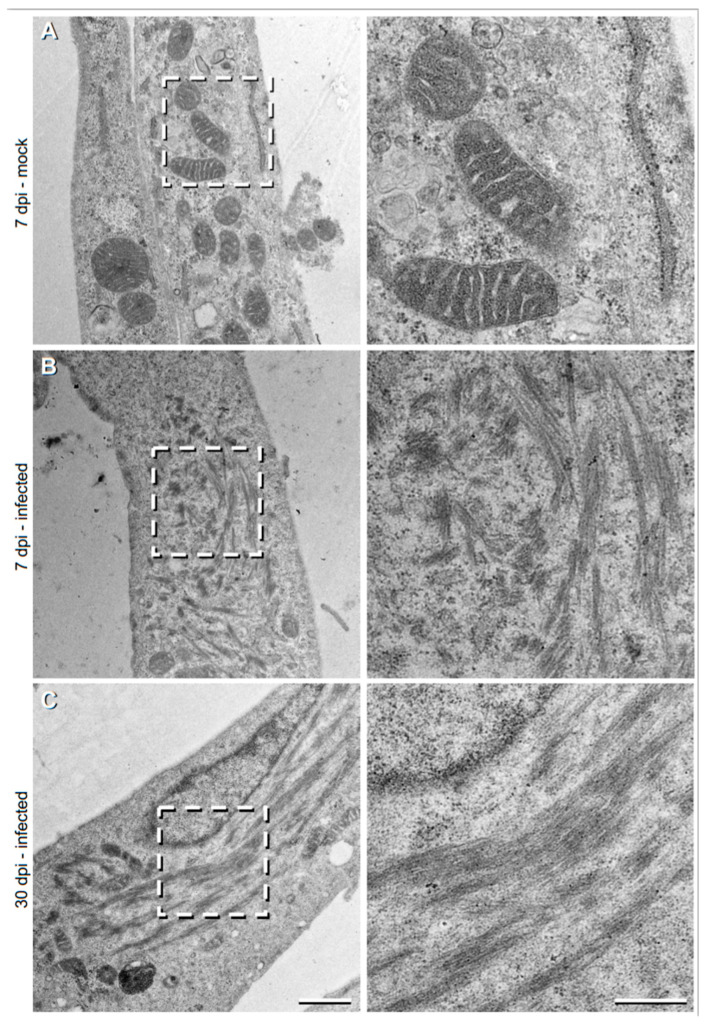
Transmission electron microscopy (TEM) images showing the filamentous structures within the TULV-infected Vero E6 cells. Vero E6 cells, (**A**) mock-infected, or infected with TULV at a MOI of 0.1 at either (**B**) 7 days post infection (dpi) or (**C**) 30 dpi. Higher magnification images from the marked inset regions are shown in the right column. Scale bars represent 500 nm and 1 μm on low and high magnification micrographs, respectively.

To further characterise these TULV NP-associated filamentous tubular structures, we then used transmission electron microscopy (TEM) to visualize the sections of TULV-infected Vero E6 cells at 36 hpi, 7 dpi and 30 dpi time points (Figure 2). During the early 36 hpi time point, no clear differences were detected between the TULV- and mock-infected cells (data not shown), most likely due to the relatively small size of TULV NP-associated structures, previously viewed (Figure 1). However, after 7 days, long filamentous and tubular structures were observed in 6% of the infected cells (eight out of 134 cells; Figure 2B), that were absent in non-infected cells, with no filaments detected in any of 61 non-infected cells that were imaged (Figure 2A). The relatively small percentage of infected cells containing filaments is explained because the EM images represent thin sections (~100 nm thick), suggesting that the filaments are concentrated in specific locations within the infected cell. Such filaments comprised clusters of small bundles, with each comprising multiple slender filamentous structures. Filament bundles were around ~100 to 1000 nm in length and spread over a large area within the cell, forming clusters that reached up to 4 μm in length. While filaments within the bundles were aligned in similar directions, the separate clusters often protruded in different orientations.

At 30 dpi, the filamentous clusters were present, but had approximately doubled in their frequency (to 10%; out of ~60 imaged cells) and had dramatically increased in size to ~5 μm in total length (Figure 2C). Individual bundles of tubular filaments were still evident, but these were less frequently observed. Instead, the bundles were longer (500–3000 nm) and shared a similar orientation. The dimensions and overall morphology of these TULV-induced structures at both the 7 dpi and 30 dpi time points coincided with those filamentous tubular structures stained for TULV NP and visualized by light microscopy, shown above (Figure 1).

### 3.3. Co-Localization of TULV NP with Cytoskeletal Components

Previous studies have shown that HTNV NP associates with actin [15], tubulin and vimentin [18], the latter forming cages surrounding HTNV NP in the vicinity of the ER-Golgi intermediate compartment (ERGIC). Consequently, we then determined the distribution of the punctate and filamentous forms of TULV NP in relation to these cytoskeletal components.

**Figure 3:**
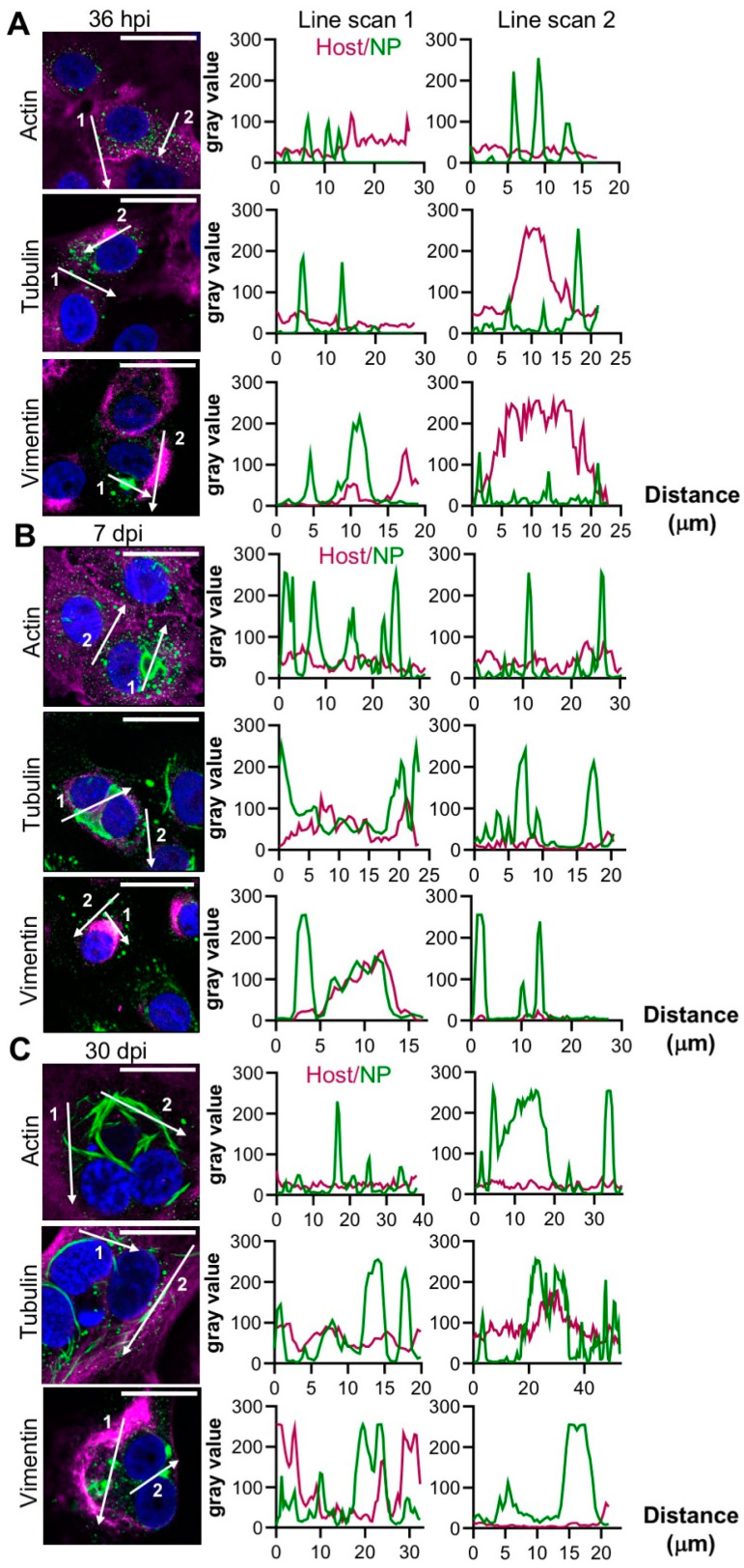
Co-localisation between the TULV NP and cytoskeletal filaments at the early, peak and persistent stages of TULV infection in Vero E6 cells. The spatial distribution of the cytoskeletal markers actin, tubulin and vimentin (magenta) was observed alongside TULV NP (green) in Vero E6 cells infected with TULV at a MOI of 0.1 by confocal microscopy at 40× magnification at (**A**) 36 h post infection (hpi), (**B**) 7 days post infection (dpi), and (**C**) 30 dpi. The scale bar represents 30 μm. Fluorescent line scans were taken using Fiji software with the designated number labels corresponding to adjacent line scan plots. Nuclei were stained with DAPI (blue), the TULV NP was detected using NP antisera and the cytoskeleton was detected using primary antibodies against β-actin, β-tubulin and vimentin. Images shown in panels A, B and C show the representative analysis of 87, 109 and 58 cells, respectively.

At 36 hpi, no association of either punctate or filamentous NP with actin, tubulin, or vimentin were observed (Figure 3A). However, at 7 dpi, vimentin was redistributed and co-localized with NP in the larger filamentous/tubular structures (Figure 3B, line scan 1) although vimentin/NP co-localization was less apparent in punctate compartments (Figure 3B, line scan 2). At 30 dpi, vimentin appeared to surround the NP structures (Figure 3C). Neither actin nor tubulin co-localized with NP at any time point. 

To further characterise the association between vimentin and NP, the infected cells were treated with okadaic acid (OA), an inhibitor of protein phosphatase 1 and 2A [24] that promotes vimentin disassembly [25]. OA treatment led to the disappearance of the typical NP structures, which rapidly reformed upon OA removal (Figure S1). In addition, OA treatment of TULV-infected cultures at 30 dpi led to reduced TULV genome copy numbers, suggesting that the disruption of the vimentin network was detrimental to TULV RNA replication (Figure S2). In contrast, nocodazole (NOC) caused no changes to the tubulin networks (Figure S1) and did not influence TULV replication (Figure S2).

### 3.4. Stress Granules (SGs) Are Recruited to Filamentous Structures during TULV Infection

PBs and stress granules (SGs) are non-membrane bound, liquid phase compartments that have been shown to stimulate the RNA synthesis of some negative sense RNA viruses and closely associate with their corresponding replication factories [26,27,28,29]. To examine the possible association of TULV NP and these compartments, the infected cells were stained with the PB marker DCP1a, a PB-resident mRNA de-capping enzyme, or the SG marker TIA-1, an RNA binding protein that scaffolds SG formation.

**Figure 4:**
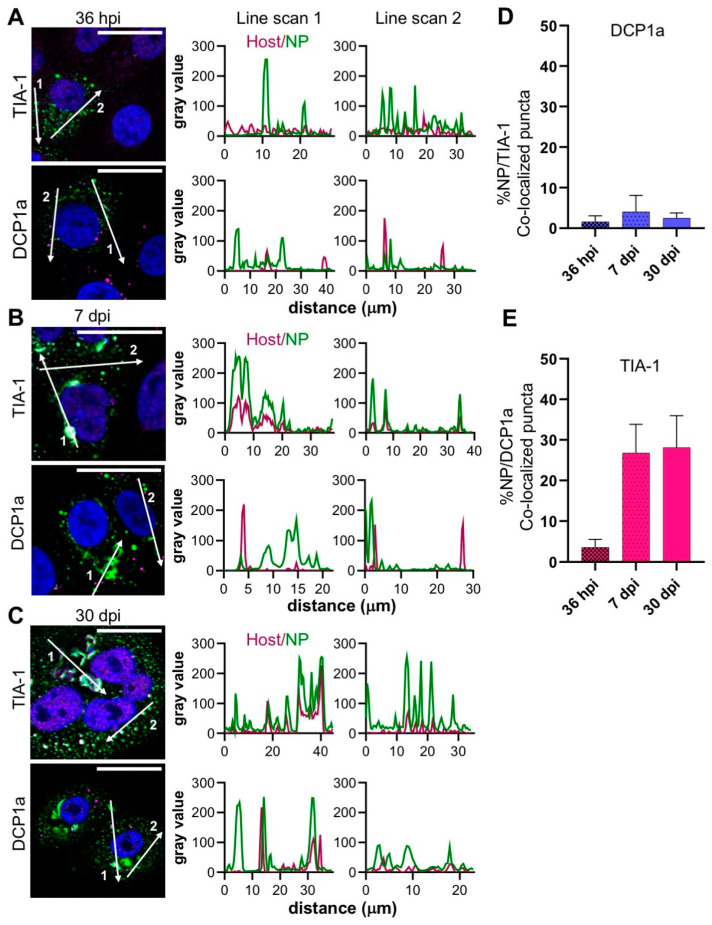
Co-localisation between TULV NP and either the stress granules (SGs) or the processing bodies (PBs) at the early, peak and persistent stages of TULV infection in Vero E6 cells. The spatial distribution of the SG marker TIA-1 and the PB marker DCP1a (magenta) was observed alongside TULV NP (green) in Vero E6 cells infected with TULV at a MOI of 0.1 by confocal microscopy at 40× magnification at the following time points; (**A**) 36 h post infection (hpi), (**B**) 7 days post infection (dpi) and (**C**) 30 dpi. The scale bars represent 30 μm. Fluorescent line scans were taken using Fiji software with the designated number labels corresponding to the adjacent line scan plots. Nuclei were stained with DAPI (blue), TULV NP was detected using NP antisera, and the SGs and PBs were detected using specific antisera against TIA-1 and DCP1a, respectively. Percentage co-localization of D) TIA-1 and E) DCP1a puncta co-localizing with NP at all three time points were quantified and represented as separate histograms. Images shown in panels A, B and C show a representative analysis of 7, 25 and 23 cells, respectively.

At the early, peak and persistent stages of the time course visualized, DCP1a-stained puncta were abundant (Figure 4A), but were spatially-distinct from the large filamentous/tubular NP-stained structures (Figure 4B,C) and with fewer than 4% co-localizing with NP (Figure 4D).

In contrast, the staining pattern of TIA-1 was different; at 36 hpi, only low numbers of TIA-1 puncta were detectable and their co-localization with NP was relatively low (Figure 4A). However, at 7 dpi and 30 dpi, the abundance of TIA-1 increased in dense cytoplasmic structures that co-localized with both filamentous/tubular NP compartments, as well as NP puncta (Figure 4B–C). The line scans associated with these later time points clearly revealed an overlap of the TIA-1 signal with both the filamentous/tubular and punctate NP signals, with their peak intensities coinciding (Figure 4B,C), corroborated by almost 30% of the TIA-1-stained compartments co-localizing with NP puncta (Figure 4E). Taken together, these results suggest that SG formation is simulated during the later stages of TULV infection, and that SGs are recruited to NP positive filamentous/tubular compartments.

### 3.5. TULV NP Associates with the Golgi during Infection

For many hantaviruses of both OW and NW clades, their corresponding NPs accumulate at sites within and adjacent to the Golgi, suggesting that this compartment may play an important role in hantavirus RNA synthesis. To better understand the intracellular distribution of TULV NP, the proximity of known ER and Golgi markers were assessed relative to NP in TULV-infected cells.

**Figure 5:**
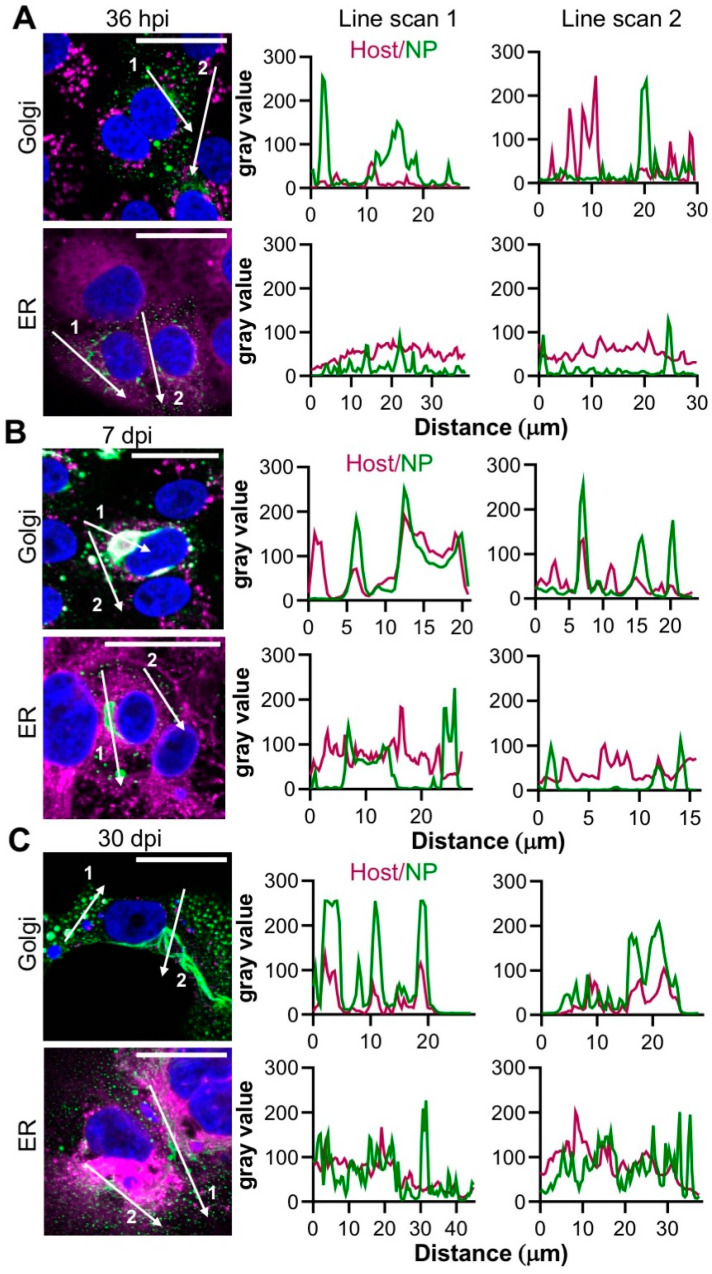
Co-localisation between the TULV NP and either the endoplasmic reticulum (ER) or the Golgi compartments at the early, peak and persistent stages of TULV infection in Vero E6 cells. The spatial distribution of the endoplasmic reticulum and the Golgi network (both magenta) was observed alongside TULV NP (green) in Vero E6 cells infected with TULV at an MOI of 0.1 by confocal microscopy at 40 X magnification at (**A**) 36 h post infection (hpi), (**B**) 7 days post infection (dpi) and (**C**) 30 dpi. The scale bars represent 30 μm. Fluorescent line scans were taken using Fiji software with the designated number labels corresponding to the adjacent line scan plots. Nuclei were stained with DAPI (blue), TULV NP was detected using NP antisera, with the ER detected using concanavalin-A and the Golgi was detected by specific antisera. Images shown in panels A, B and C show a representative analysis of the 19, 27 and 13 cells, respectively.

At 36 hpi, the Golgi appeared with the classical morphology of elongated and stacked vesicular compartments that were distinct from NP (Figure 5A). In contrast, at both 7 dpi and 30 dpi, there was extensive overlap with NP and the Golgi, in both large filamentous/tubular NP structures, and also punctate NP, with closely coinciding peaks on the associated line scans (Figure 5B,C). No such co-localisation between NP and the ER marker concanavalin A was observed at any of the specified time points (Figure 5A–C). In addition, NP showed minimal co-staining with clathrin, Rab5, Rab7, Rab11 or LAMP 1 markers, implying that the punctate NP structures were not endosomal in nature (Figure S3).

### 3.6. Products of TULV RNA Synthesis co-Localize with NP

The results presented thus far show that NP exhibits a close co-localization with the Golgi and SGs, within large filamentous and tubular structures. As NP is the major protein component of TULV RNPs, which act as templates for TULV mRNA transcription and RNA replication, we next investigated whether these NP-associated compartments were also stained for the products of TULV RNA synthesis. 

Two independent FISH probe sets were designed comprising multiple non-overlapping fluorescently labelled short oligonucleotides with near complete coverage of the TULV S segment RNA target. One set was designed to detect negative sense TULV S segment RNAs, comprising the vRNA genome alone, whereas a second set was designed to detect positive sense S segment RNAs. The positive sense probe set would unavoidably hybridize to both S segment mRNAs and cRNAs due to their nested genetic organisation, however, as most positive sense RNAs generated during bunyavirus infection are mRNA transcripts [30], the majority of the detected positive sense signal could be attributed to mRNA transcripts, with only a minor contribution from cRNA replication products.

At the early, peak and persistent time points, the cells were fixed and stained for TULV NP along with the individual probe sets. Only the cells staining positive for NP were also stained with the FISH-probe sets, showing that the probes were specific for viral RNA targets. Interestingly, the ability of the probe sets to detect negative sense RNAs showed that the association of NP with RNA within assembled RNPs did not prevent probe binding. In addition, the TULV RNA signal was not detected outside regions positive for NP, strongly suggesting that, as expected, NP is required for all TULV RNA synthesis activities.

**Figure 6:**
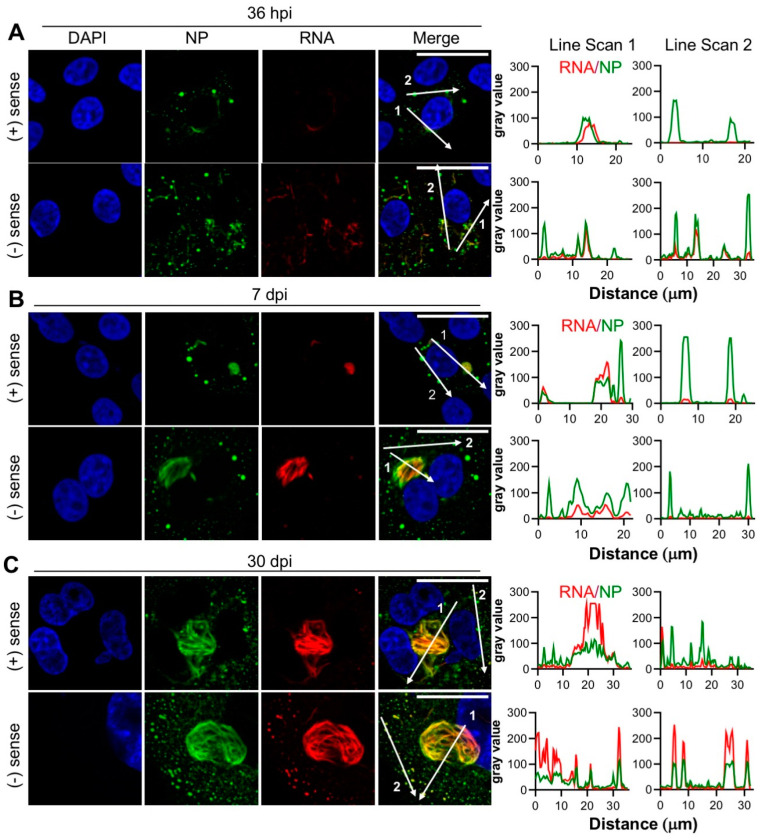
Co-localisation between the TULV NP and the TULV small (S) segment RNAs at the early, peak and persistent stages of TULV infection in Vero E6 cells. The spatial distribution of the TULV NP (green) with positive and negative sense S segment RNA (red) detected with strand-specific probe sets was observed using confocal microscopy at (**A**) 36 h post infection (hpi), (**B**) 7 days post infection (dpi) and (**C**) 30 dpi. The size bars represent 30 μm. Fluorescent line scans were taken using Fiji software with the designated number labels corresponding to the adjacent line scan plots. Nuclei were stained with DAPI (blue), TULV NP was detected using NP antisera and S segment RNA was detected using sense or anti-sense RNA FISH probes. Images presented in panels A, B and C show a representative analysis of 14, 25 and 8 cells, respectively.

At 36 hpi, 7 dpi and 30 dpi time points, the signal corresponding to both the positive and negative sense RNAs co-localized with NP, with the extremely close co-localization of NP and RNA within the filamentous/tubular objects (Figure 6A–C). RNA and NP also co-localized within some NP puncta of various sizes, although with a less consistent level of abundance. Line scan analysis of the filamentous/tubular and punctate NP structures supported these observations, with consistently high levels of the NP and RNA signal within the filamentous/tubular structures, but often lower levels of RNA in NP puncta.

At all of the time points assessed, there were no consistent differences between the distribution of positive and negative sense RNAs in relation to the NP structures, suggesting that the synthesis of mRNA/cRNAs and vRNAs is not segregated.

### 3.7. Concurrent Detection of TULV and Cellular Components in Persistently Infected Cells

**Figure 7:**
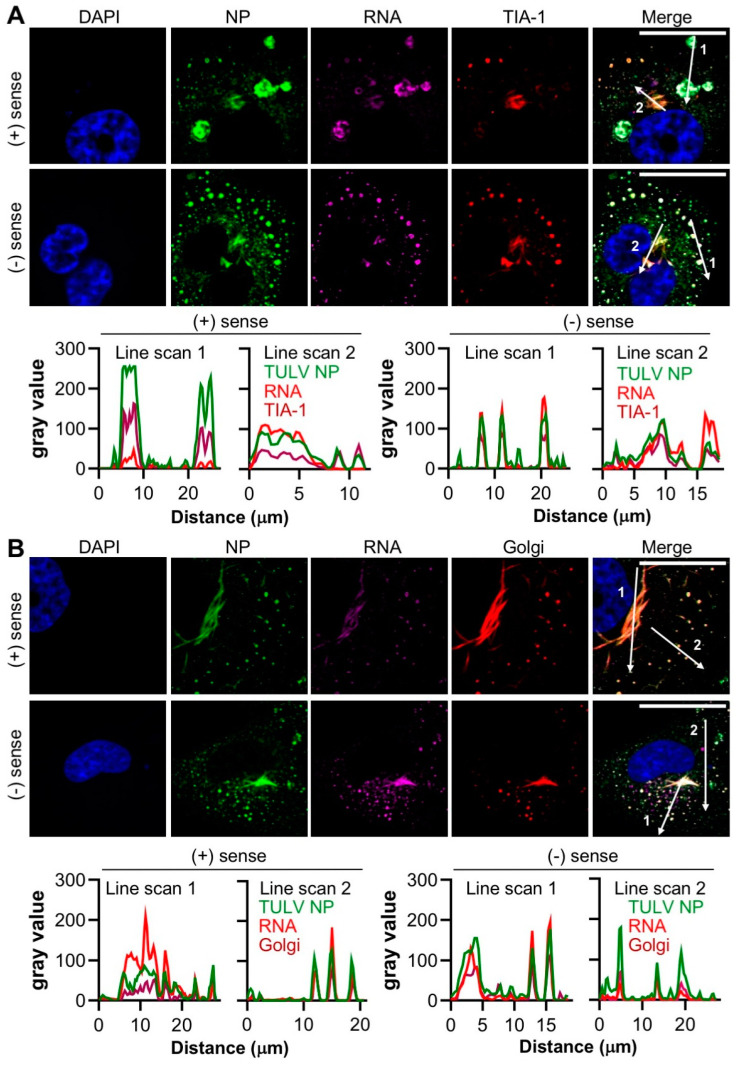
Co-localisation between the TULV NP, the TULV S segment positive and the negative sense RNAs and host cell components TIA-1 and the Golgi marker in Vero E6 cells at 30 days post infection. The spatial distribution of the TULV NP (green), the S segment RNA (magenta) and the host cell markers (**A**) TIA-1, or (**B**) Golgi (both red) were observed during the persistent infections using confocal microscopy at 40× magnification. Fluorescent line scans were taken using Fiji software with designated number labels corresponding to the adjacent line scan plots. The nuclei were stained with DAPI (blue), TULV NP was detected using NP antisera, with TIA-1 and Golgi detected using specific antisera. Scale bar represents 30 μm. TULV NP was detected using NP antisera, and S segment RNAs were detected using separate sets of positive sense or negative-sense RNA FISH probes. Images shown in panels A and B show a representative analysis of 8 and 20 cells, respectively.

To confirm the above findings, and unequivocally show that the staining of viral and host components were located within the same compartments, 30 dpi persistently infected cells were co-stained for either NP/RNA/TIA-1 or NP/RNA/Golgi, concurrently (Figure 7).

Merging the signals from all three channels representing NP/RNA/TIA-1 confirmed that the NP, TIA-1 and both polarities of TULV RNA were simultaneously present within both filamentous and punctate structures. As shown above (Figure 6), all RNA staining coincided with that of NP, however, some of these regions were devoid of TIA-1. Similarly, merging the signals for NP/RNA/Golgi revealed high levels of co-localization for all three markers within the filamentous/tubular compartments (Figure 7B). Along with the results shown in Figure 5, these findings strongly suggest that TULV infection leads to the redistribution of the Golgi to coincide with TULV RNAs, and the formation of structures that we suggest most likely represent the viral replication factories, the major sites of TULV RNA synthesis.

## 4. Discussion

Many hantaviruses establish persistent infections within their rodent hosts by manipulating the host cellular environment to allow the abundant synthesis of viral components for an extended time period, while also evading or minimizing the cellular innate immune response [6,7]. To better understand the intracellular events during long-term hantavirus infections, we used immunofluorescent microscopy to investigate the intracellular distribution of TULV NP and RNA, alongside multiple host cell components during an extended time course of up to 30 days, during which time the persistent infection by TULV has been previously demonstrated [23]. This was combined with EM imaging to understand the morphological changes that TULV induces in infected cells. Our findings revealed that as the infection progressed, the NP formed increasingly large ultrastructural assemblies, mostly with an overall filamentous and tubular appearance, but also as discrete puncta (Figure 1). At all the time points examined, these NP assemblies closely co-localized with TULV RNA, regardless of its negative or positive sense polarity (Figure 6). This close association between all hantavirus RNA and NP is consistent with the known structure of the hantavirus RNP segments, in which hantaviral vRNAs and cRNAs are always found in close association with NP, forming extended helical assemblies [31]. Interestingly, our observations also suggest that TULV mRNAs, which will likely comprise the majority of positive sense TULV-specific RNA in the infected cell [30], also remain in close proximity with NP. This may reflect the organisation of the replication factories, with newly transcribed S, M and L segment mRNAs remaining in the vicinity of their respective NP enwrapped vRNA templates. Alternatively, it may reflect the previous findings that the hantavirus NP binds the 5′ mRNA cap at a separate RNA binding site [32], and this interaction is proposed to enable mRNA circularization, so as to promote eIF4F independent translation [33]. The lack of distinction between the sites of positive sense and negative sense RNA staining at the resolution used here also suggests that TULV RNA replication and mRNA transcription processes are not physically separated into distinct compartments. 

Co-staining experiments using markers for intracellular compartments revealed that NP and TULV RNA co-localized within structures that were closely associated with the Golgi (Figure 5). At the early stages of infection (36 hpi), NP, RNA and Golgi components localized within both punctate and irregular filamentous structures within and surrounding compartments that exhibited a canonical Golgi morphology. At the later time points of 7 dpi and 30 dpi, NP and RNA were detected with increased abundance within a single filamentous/tubular structure of large dimensions, which also closely co-localized with the Golgi (Figure 5B–C). The morphology of this filamentous/tubular NP/RNA/Golgi stained structure was atypical of the Golgi compartment, with a clear loss of the typical fused compact ribbon structure associated with Golgi cisternae, suggesting that the Golgi was re-modelled, most likely by TULV-related activities. As both TULV NP and RNA components localised to these compartments (Figure 7), it is difficult to escape the conclusion that this remodelled Golgi is the site of TULV RNA synthesis and accumulation, and most likely represents TULV replication factories.

The visualization of long filamentous structures at later 7 dpi and 30 dpi time points in TULV-infected cells only, by TEM, was in agreement with our findings using light microscopy (Figure 2). These TULV-infected cell sections revealed the increased detail of the filamentous/tubular NP/RNA/Golgi stained structures, and confirmed their atypical morphology. Their apparent absence in sections of 36 hpi infected cells likely reflects their low abundance and the correspondingly high likelihood that they would not appear in the selected sections. Few studies have employed EM to study hantavirus-infected cells, with most analyses focusing on the morphology of cell-associated hantavirus particles [34,35,36]. However, the filamentous structures we observed here are reminiscent of filamentous and granular structures induced by the Sin Nombre virus [21], which were also in close proximity to the Golgi. However, in this study we showed that these filamentous structures also harbour abundant viral RNA.

If the filamentous/tubular NP-stained structures are the sites of RNA synthesis and accumulation, then the function of the punctate NP structures that are seen throughout the infection time course remains unclear. As they mostly stain weakly for RNA, we propose that they are unlikely to be sites of on-going abundant RNA synthesis. One possibility is that they may represent Golgi-derived particles, released by Golgi fragmentation, although an alternative possibility that we cannot rule out, is that the smallest of these structures may represent exocytic vesicles containing assembled viruses in transit towards the plasma membrane for release.

In contrast to the SG marker TIA-1, the levels of co-localization between the NP and the PB marker DCP1a were considerably lower (Figure 4), and this was unexpected given the previous reports of a close association between DCP1a and the NP of the NW SNV, where NP is proposed to bind and protect capped RNAs for later delivery to the transcribing RdRp [9]. This may represent fundamental differences between the host–pathogen interactions of NW and OW hantavirus species, and may suggest that OW hantaviruses such as TULV do not acquire or process host cell cap moieties in the same way. Given the high degree of association we report between TULV NP and TIA-1, an alternative cap resource may be SGs.

The observation that the SG marker TIA-1 showed a punctate distribution at the early stages of virus infection, but was filamentous/tubular distribution at later times, suggests that it was re-distributed into the Golgi-derived filamentous/tubular compartments (Figure 4A–D). To the best of our knowledge, SGs and the Golgi have not been previously reported to extensively co-localize for a native function, and thus we propose that this redistribution is in response to TULV infection. Whether this represents a pro-viral or anti-viral response is currently unknown. Considerable evidence suggests that SGs represent innate immune signalling platforms [37] and one possibility is that their sequestration into TULV replication factories may prevent or delay a host cell anti-viral response to infection. Such a mechanism of immune evasion may contribute to the establishment of the characteristic state of hantavirus persistence. 

## Supplementary Materials

The following are available online at https://www.mdpi.com/2073-4409/9/7/1569/s1, Table S1. DNA oligonucleotide probes designed to hybridize with TULV S segment RNAs in FISH experiments. Sequences shown are written 5′ to 3′ and represent the actual probe sequences, Movie S1. TULV-infected Vero E6 cells at 30 dpi were stained using TULV NP antisera and DAPI, and successive Z plane images taken, which were compiled into a movie, with rotation about a single axis, Figure S1. The effect of cytoskeletal disassembly and reorganisation on the localisation of TULV NP in Vero E6 cells persistently infected with TULV. Vero E6 cells were infected with TULV at an MOI of 0.5, and at 30 dpi cytoskeleton filaments were disassembled by treatment with A) 400 nM okadaic acid (OA) for periods of 60 and 90 min. Distribution of vimentin and TULV NP were examined using LSCM at 40 X magnification, stained with specific antisera NP antisera (green) and vimentin (magenta) with nuclei stained with DAPI (blue). Vimentin filaments were allowed to reassemble by the removal of OA, and the distribution of NP and vimentin examined as above. The scale bar represents 30 μm. B) The effect of microtubule depolymerisation on TULV NP localisation was also examined in Vero E6 cells at 30 dpi. Microtubules were depolymerised by treating Vero E6 cells with 17 μM nocodazole (NOC) for 60 min, with the distribution of tubulin and TULV NP examined using LSCM at 40 X magnification, stained with specific antisera NP antisera (green) and tubulin (magenta) with nuclei stained with DAPI (blue). The scale bar represents 30 μm, Figure S2. Okadaic acid treatment of TULV-infected Vero E6 cells decreases viral genome copies. TULV-infected Vero E6 cells at 30 dpi were treated with 400 nM okadaic acid (OA) for 30, 60 or 90 min or 17 μM nocodazole (NOC) for 60 min. After harvesting, the TULV genome copies in cell lysates were quantified by qRT-PCR., Figure S3. Co-localisation of cellular trafficking proteins with TULV NP in TULV-infected Vero E6 cells during early and persistent time points. The spatial distribution of Rab5, Rab7, Rab11, LAMP1 and clathrin (magenta) was observed alongside TULV NP (green) in TULV-infected Vero E6 cells at A) 36 hpi and B) 30 dpi by LSCM at 40 X magnification using specific antisera against TULV NP, Rab5, Rab7, Rab11, LAMP1 and clathrin. Nuclei are stained with DAPI (blue). The scale bar represents 30 μm. Fluorescent line scans taken using Fiji software.
